# Neuronal Transmembrane Chloride Transport Has a Time-Dependent Influence on Survival of Hippocampal Cultures to Oxygen-Glucose Deprivation

**DOI:** 10.3390/brainsci9120360

**Published:** 2019-12-06

**Authors:** Ana-Maria Zagrean, Ioana-Florentina Grigoras, Mara Ioana Iesanu, Rosana-Bristena Ionescu, Diana Maria Chitimus, Robert Mihai Haret, Bogdan Ianosi, Mihai Ceanga, Leon Zagrean

**Affiliations:** 1 Neuroscience Laboratory, Division of Physiology and Neuroscience, Carol Davila University of Medicine and Pharmacy, Bucharest 050474, Romania; grigorasif@gmail.com (I.-F.G.); iesanu_mara@yahoo.com (M.I.I.); bristena.ionescu@gmail.com (R.-B.I.); chitimus.diana@yahoo.com (D.M.C.); robert.haret@stud.umfcd.ro (R.M.H.); ianosi.bogdan@gmail.com (B.I.); leon.zagrean@umfcd.ro (L.Z.); 2 Wellcome Centre for Integrative Neuroimaging, FMRIB, Nuffield Department of Clinical Neurosciences, University of Oxford, Oxford OX3 9DU, UK; 3 Department of Neurology, Division of Neurocritical Care, Medical University of Innsbruck, Innsbruck 6020, Austria; 4 Hans Berger Department of Neurology, Jena University Hospital, Jena 07747, Germany

**Keywords:** oxygen-glucose deprivation, hippocampal culture, NKCC1, KCC2, GABA_A_, stroke

## Abstract

Neuronal ischemia results in chloride gradient alterations which impact the excitatory–inhibitory balance, volume regulation, and neuronal survival. Thus, the Na^+^/K^+^/Cl^−^ co-transporter (NKCC1), the K^+^/ Cl^−^ co-transporter (KCC2), and the gamma-aminobutyric acid A (GABA_A_) receptor may represent therapeutic targets in stroke, but a time-dependent effect on neuronal viability could influence the outcome. We, therefore, successively blocked NKCC1, KCC2, and GABA_A_ (with bumetanide, DIOA, and gabazine, respectively) or activated GABA_A_ (with isoguvacine) either during or after oxygen-glucose deprivation (OGD). Primary hippocampal cultures were exposed to a 2-h OGD or sham normoxia treatment, and viability was determined using the resazurin assay. Neuronal viability was significantly reduced after OGD, and was further decreased by DIOA treatment applied during OGD (*p* < 0.01) and by gabazine applied after OGD (*p* < 0.05). Bumetanide treatment during OGD increased viability (*p* < 0.05), while isoguvacine applied either during or after OGD did not influence viability. Our data suggests that NKCC1 and KCC2 function has an important impact on neuronal viability during the acute ischemic episode, while the GABA_A_ receptor plays a role during the subsequent recovery period. These findings suggest that pharmacological modulation of transmembrane chloride transport could be a promising approach during stroke and highlight the importance of the timing of treatment application in relation to ischemia-reoxygenation.

## 1. Introduction

Stroke is the second leading cause of mortality worldwide, with an annual 5.5 million death toll [1]. Moreover, the incidence of stroke in young adults (18–50 years) has significantly increased in the past decade, with considerable socioeconomic impact because of high health-care costs and loss of labor productivity [2]. Despite major efforts to advance knowledge on the pathophysiology and treatment of this severe medical disorder, we still face a paucity of effective therapeutic protocols. Clinical practice reveals a preclinical setting, during which cerebral ischemia continues, and for which there is currently no approved treatment, as well as a clinical therapeutic window during which revascularization may be attempted. Present guidelines recommend pharmacological or interventional revascularization strategies, which are limited by a time window and have a non-insignificant degree of treatment failure or complication rate [3,4,5]. During acute treatment, patients often receive pharmacological treatments for sedation (e.g., during transport in the preclinical phase, when undergoing interventional procedures, or during intensive care treatment) or for increased intracranial pressure (e.g., after a malignant stroke or intracerebral hemorrhage), which involve modulation of gamma-aminobutyric acid A (GABA_A_) receptors or chloride transporters [6,7,8]. It is yet to be determined how these drugs and the timing of their application (preclinical, during ischemia, or after revascularization) influence neuronal viability. 

Ischemic stroke involves the disruption of the aerobic metabolism on which neurons strictly function [9], which eventually leads to cellular death due to a decrease in ATP levels and ionic imbalance across the neuronal cell membrane. During this process, the excitatory–inhibitory balance is dysregulated, which may accelerate neuronal demise [10,11,12]. Gamma-aminobutyric acid (GABA) has a crucial role in counteracting neuronal excitation, mainly acting through the activation of GABA_A_ receptors, the effect depending on the chloride transmembrane gradient. Chloride homeostasis in the brain is primarily regulated by two SLC12A cation-chloride co-transporters: K^+^/ Cl^−^ co-transporter KCC2, which extrudes Cl^−^ and K^+^ [13], and the Na^+^/K^+^/Cl^−^ co-transporter NKCC1, which shuttles Cl^−^, K^+^, and Na^+^ into the neuron, using the electrochemical gradient of Na^+^ [14]. These two transporters are responsible for regulating most of the [Cl^−^]_i_, which in turn sets the strength and polarity of the GABAergic transmission in the brain. During development, KCC2 expression is upregulated, while NKCC1 levels are decreasing, leading to a lower [Cl^−^]_i_. KCC2 mRNA expression in the rat brain is minimal at birth and increases in early postnatal life, changes that are accompanied by a reduction in NKCC1 expression and are similar to those observed in the developing human brain [15]. These characteristic changes during neurodevelopment are often referred to as the excitatory-to-inhibitory GABA switch [16]. During the ischemic process, there are several alterations in the expression and activity of cation-chloride transporters, that lead to a higher [Cl^−^]_i_. Thus, KCC2 mRNA and protein levels are significantly downregulated in rats after ischemia [17,18], in a process involving the endogenous brain-derived neurotrophic factor (BDNF) and tyrosine kinase B (TrkB) receptors [19,20,21]. This contributes to the disruption of Cl^−^ extrusion, leading to its accumulation after oxygen-glucose deprivation (OGD) and a diminished GABAergic inhibition [17]. On the other hand, NKCC1 expression during ischemia remains unchanged, while its activity increases through phosphorylation of its threonine residues [22]. NKCC1 activation evokes a pathway that involves the Raf/MEK/ERK cascade, cAMP response element-binding protein (CREB) phosphorylation, and hypoxia-inducible factor HIF-1α upregulation that causes an elevation in vascular endothelial growth factor (VEGF) expression and the subsequent induction of neurogenesis [23]. The reversal of NKCC1/KCC2 ratio decreases the inhibitory strength of GABAergic transmission, leading to pathological states.

In summary, chloride transporters’ activity, during different brain developmental stages and pathological conditions, impacts the neuronal excitability and response to ischemic insult, suggesting them as potential targets for neuroprotection paradigms. As such, preserving a low [Cl^−^]_i_ could be associated with higher cellular viability. We have previously shown that chloride transport modulation in immature hippocampal cultures has a neuroprotective effect both *in vitro* during OGD [24], as well as in an *in vivo* model of perinatal asphyxia [25]. Considering that mature neurons display altered chloride gradients under stroke conditions, which may also be influenced by many pharmaceutical agents used in stroke patients, there is an increased interest to determine the potentially favorable or detrimental effects of modulating chloride gradients.

In the present study, we aimed to examine the effect of specific blocking of NKCC1 (bumetanide) [26], KCC2 (R(+)-[(dihydroindenyl)oxy] alkanoic acid—DIOA) [27], and GABA_A_ receptor (gabazine) [28] as well as the effect of the GABA_A_ agonist isoguvacine, on the viability of hippocampal cultures undergoing OGD. Viability was measured using a metabolic assay which requires a concomitant reoxygenation time of 3 h. We measured the effect of drug application both during OGD (mimicking the preclinical therapeutic window), as well as post-exposure (mimicking the revascularization). Considering the role that [Cl^−^]_i_ plays in both volume regulation and excitatory–inhibitory balance, we postulated that modulating [Cl^−^]_i_ by blocking its transmembrane transport would influence cellular viability with an inversely proportional relation. Our results suggest a complex, timing-dependent effect of treatment on cellular viability.

## 2. Materials and Methods

### 2.1. Primary Cultures of Hippocampal Neurons

Primary hippocampal cell cultures were obtained from Wistar rat pups at postnatal day zero (P0), following a protocol previously described [29]. All animal procedures were carried out with the approval of the local ethics committee for animal research Carol Davila University of Medicine and Pharmacy (Bucharest, Romania) and in accordance with the European Communities Council Directive 86/609/EEC.

Briefly, the hippocampi were dissected, and the meninges and vascular plexuses were removed. The tissue was subjected to mechanical trituration and enzymatic digestion using papain (Worthington, 3.8 U/mL), and the cell suspension obtained was kept in culture medium (CM) containing Neurobasal-A medium (Invitrogen) supplemented with B-27 (Invitrogen), l-glutamine (HyClone, 0.5 mM), and antibiotic-antimycotic (Invitrogen). The cell suspension was plated in 24-well plates coated with poly-d-lysine (Sigma, 70,000–150,000 kDa, 100 µg/mL) at a density of 150,000 cells/well. The plates were placed in a humidified 5% CO_2_ incubator at 37 °C. After 5 days *in vitro* (DIV), the cultures were fed by removing half of the CM volume in each well (250 µL) and replacing it with 400 µL newly prepared CM.

### 2.2. Exposure to Oxygen-Glucose Deprivation (OGD)

The primary hippocampal cell cultures were grown until DIV 7, a developmental time accompanied in rodents with a progressive switch of the GABA_A_ receptor activation from excitatory to inhibitory [30]. Previous *in vitro* experiments in mice reported that at DIV 6 the NKCC1 expression is approximately 4 times lower than at DIV 1, while the KCC2 expression is increased by approximately 15 times compared to DIV 1, which is consistent with a mature phenotype [31].

OGD was induced on DIV 7 as previously described [24,32], by removing CM from the wells and replacing it with a deoxygenated experimental medium without glucose (EM-G), consisting of Neurobasal-A without both glucose and sodium pyruvate (Life Technologies), supplemented with HEPES (Sigma, 10 mM) and l-glutamine (HyClone, 0.5mM). The plates were then immediately placed in a humidified hypoxia chamber (Billups-Rothenberg), which was flushed with 100% N_2_ for 10 min, then sealed, and placed in an incubator at 37 °C for 2 h.

Afterwards, EM-G was removed and cultures were gently washed with an assessment medium (AM) containing Neurobasal-A without phenol red (Life Technologies), supplemented with HEPES (10 mM) and l-glutamine (HyClone, 0.5 mM) and incubated for 3 h at 37 °C and 5% CO_2_ in AM with resazurin (Sigma, 100 µM) to assess their metabolic viability. Control cultures were subjected to a sham exposure to normoxic conditions at 37 °C and 5% CO_2_ for 2 h in an experimental medium containing Neurobasal-A with glucose 25 mM (EM + G).

### 2.3. Assessment of Cellular Metabolism and Viability

The viability of the hippocampal cultures was evaluated by measuring their cellular metabolism using a cell permeable redox indicator, resazurin (Aldrich) [33,34], as previously described [24]. Within cells with active metabolism, resazurin is reduced to resorufin, a fluorescent product. Resazurin requires a 3 h reoxygenation period to be metabolized, allowing for a delayed, post-exposure treatment time window. Resorufin fluorescence was read at 535 nm excitation and 595 nm emission using a multimode detector (DTX880, Beckman Coulter, Inc., Indianapolis, IN, USA). 

### 2.4. Treatment with Chloride Membrane Transport Blockers

Treatments were applied either during the 2 h exposure to OGD/sham normoxia (T1) or post-exposure, during the 3 h necessary for viability assessment (T2). 

**Figure 1:**
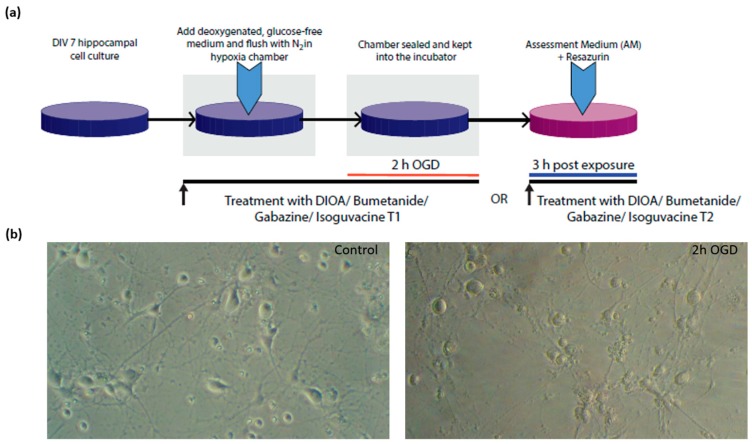
(**a**) Experimental design. (**b**) Phase-contrast microscopy of control and OGD-exposed DIV 7 hippocampal cell cultures. DIV—days *in vitro*, OGD—oxygen-glucose deprivation, DIOA—R(+)-[(dihydroindenyl)oxy] alkanoic acid, T1—treatment applied during OGD, T2—post-exposure treatment.

We blocked outward Cl^−^ transport with DIOA, a KCC2 blocker (Sigma, 20 µM), and inward Cl^−^ transport with bumetanide (Sigma, 10 µM), a NKCC1 blocker as a means for increasing, respectively decreasing [Cl^−^]_i_ either during OGD (T1) or post-exposure (T2). We also used the GABA_A_ receptor antagonist gabazine (Sigma, 30 µM) as well as the GABA_A_ receptor agonist isoguvacine (Sigma, 40 µM) to modulate the GABA_A_ channel, both during OGD (T1) and during post-exposure (T2). All treatments were also applied to normoxic cultures. Experimental design and treatment times are illustrated in Figure 1.

### 2.5. Data Analysis

The data was analyzed using Prism (GraphPad 7 Software). Data is presented as percentages normalized to the control values (normoxia + no treatment condition). The statistical unit was the number of wells tested per condition (n). The viability of cultures after different OGD treatments was compared to that of OGD without treatment from the same cultures. This results in slightly different OGD viabilities across treatment conditions. However, the degree of metabolic lesion was severe in all cultures, ranging from 42.5% to 59.6% (average 52.25%). All variables were tested for normality of distribution using the Shapiro–Wilk test and central tendencies are consequently reported as mean ± SD. Statistical significance was evaluated using ordinary two-way analysis of variance (ANOVA) with factors of oxygenation (normoxia, OGD) and treatment (no treatment, treatment T1, treatment T2) and Tukey’s multiple comparisons test, with α set at 0.05.

## 3. Results

### 3.1. DIOA Treatment during Oxygen-Glucose Deprivation Decreases Cellular Viability of DIV 7 Hippocampal Cell Cultures

**Figure 2:**
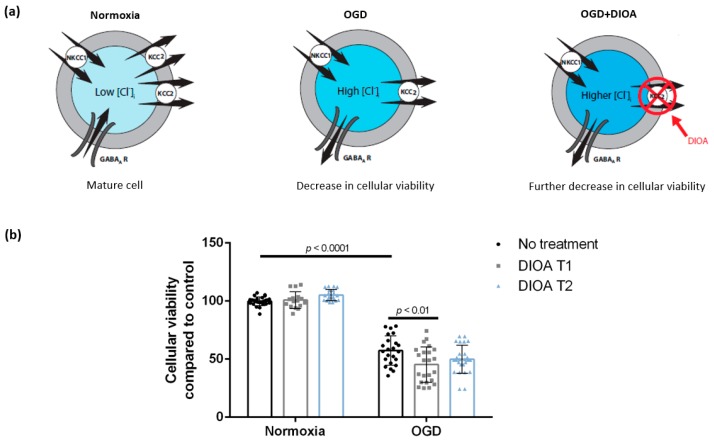
DIOA treatment during OGD is associated with a further decrease in cellular viability of DIV 7 hippocampal neurons. (**a**) Hypothesis: blocking KCC2 using DIOA may aggravate intracellular Cl^−^ accumulation following ischemia-induced KCC2 downregulation [18]. (**b**) Cellular viability of DIV 7 hippocampal neurons decreased after 2 h of OGD. DIOA treatment during OGD (DIOA T1) but not post-exposure (DIOA T2) further decreased cellular viability. Bars represent mean ± SD. OGD—oxygen-glucose deprivation, NKCC1—the Na^+^/K^+^/Cl^−^ co-transporter, KCC2—the K^+^/Cl^−^ co-transporter, GABA_A_ R—the gamma-aminobutyric acid A receptor, [Cl^−^]_i_—intracellular chloride concentration, DIOA—R(+)-[(dihydroindenyl)oxy] alkanoic acid.

Our 2 h OGD protocol decreased neuronal viability to 57.3% ± 12.6% (*n* = 23, six cultures) as measured by the resazurin assay. Application of DIOA further decreased cellular viability when applied during OGD (45.3% ± 15.2%, *n* = 23, six cultures), but had no effect when applied post-exposure (49.8% ± 12.1%, *n* = 26, six cultures). Cellular viability of normoxia cultures treated with DIOA at T1 and T2 was 100.8% ± 7.2% (*n* = 16, five cultures) and 105.1% ± 4.8% (*n* = 20, six cultures), respectively (Figure 2). There was a significant main effect of OGD (F (1,128) = 787.3, *p* < 0.0001) and DIOA treatment (F (2,128) = 3.283, *p* = 0.0407) as well as a significant OGD–DIOA interaction (F (2,128) = 5.851, *p* = 0.0037). After Tukey’s multiple comparisons, we found a significant difference between OGD and OGD + DIOA T1 (mean difference = 12.03, 95% CI = (3.162; 20.9), *p* = 0.0019), but no significant difference between OGD and OGD + DIOA T2 or between any of the normoxic treatment conditions.

### 3.2. Treatment with Bumetanide during Oxygen-Glucose Deprivation Is Associated with Increased Cellular Viability of DIV 7 Hippocampal Cells

**Figure 3:**
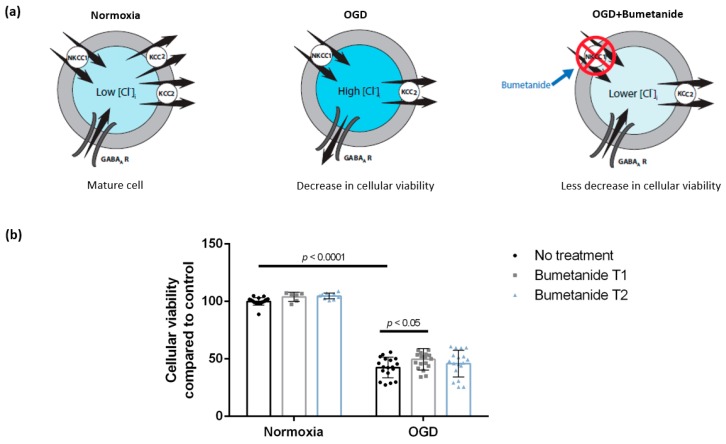
Bumetanide treatment during OGD is associated with a higher viability of DIV 7 hippocampal cell cultures. (**a**) Hypothesis: Blocking NKCC1 using bumetanide may compensate the intracellular Cl^−^ accumulation following ischemia-induced KCC2 downregulation [18]. (**b**) Cellular viability of DIV 7 hippocampal neurons decreased after 2 h of OGD. Bumetanide treatment during OGD (Bumetanide T1), but not post-exposure (Bumetanide T2), increased cellular viability. Bars represent mean ± SD. OGD—oxygen-glucose deprivation, NKCC1—the Na^+^/K^+^/Cl^−^ co-transporter, KCC2—the K^+^/Cl^−^ co-transporter, GABA_A_ R—the gamma-aminobutyric acid A receptor, [Cl^−^]_i_—intracellular chloride concentration.

Treatment with bumetanide, an NKCC1 blocker, resulted in an increase of cellular viability from 42.5% ± 8.9% (*n* = 18, 5 cultures, OGD with no treatment) to 49.6% ± 9.4% (*n* = 18, 5 cultures) when applied during OGD (Bumetanide T1). However, bumetanide administered post-exposure had no effect on cellular viability (45.9% ± 11.6%, *n* = 18, four cultures, Bumetanide T2) (Figure 3). Viability of sham wells, which were kept for 2 h in normoxia and treated with Bumetanide at T1 and T2, was 103.9% ± 4% (*n* = 6, two cultures) and 104.7% ± 2.6% (*n* = 10, three cultures), respectively. There was a significant main effect of OGD (F (1,85) = 935.7, *p* < 0.0001) and bumetanide treatment (F (2,85) = 3.635, *p* = 0.0306) but no significant OGD–bumetanide interaction (F (2, 85) = 0.4370, *p* = 0.6474). After Tukey’s multiple comparisons, we found a significant difference between OGD and OGD + Bumetanide T1 (mean difference = −7.135, 95% CI = (−13.533; −0.736), *p* = 0.0251), but no significant difference between OGD and OGD + Bumetanide T2 or between any of the normoxic treatment conditions.

### 3.3. Blocking the GABA_A_ Receptor Using Gabazine Post-Exposure, but Not during Oxygen-Glucose Deprivation, Is Associated with Decreased Cellular Viability of DIV 7 Hippocampal Cells

**Figure 4:**
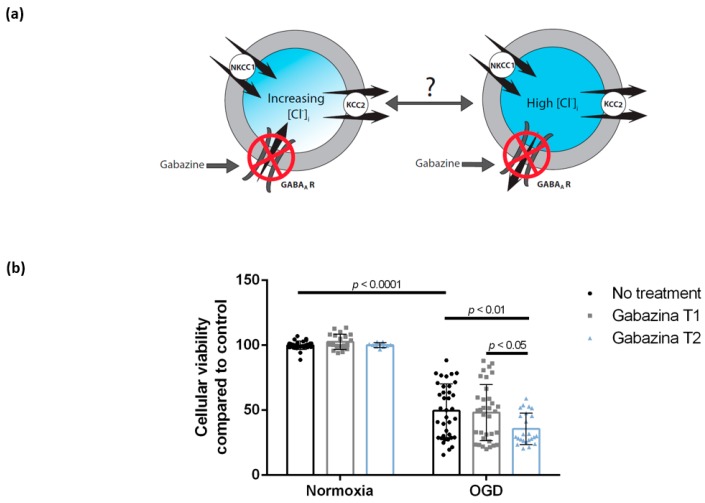
Gabazine treatment post-exposure, but not during OGD, is associated with a decrease in the viability of DIV 7 hippocampal neurons. (**a**) Hypothesis: The direction of Cl^−^ transmembrane flow following GABA_A_ receptor activation depends on [Cl^−^]_i_ and its reversal potential. Blocking of GABA_A_ receptors with gabazine during or following OGD can therefore either block the inflow or the outflow of chloride ions. (**b**) Cellular viability of DIV 7 hippocampal neurons decreased after 2 h of OGD. Cellular viability of DIV 7 hippocampal neurons after gabazine treatment post-OGD exposure (Gabazine T2) is lower than either OGD alone or gabazine treatment during OGD (Gabazine T1). Bars represent mean ± SD. OGD—oxygen-glucose deprivation, NKCC1—the Na^+^/K^+^/Cl^−^ co-transporter, KCC2—the K^+^/Cl^−^ co-transporter, GABA_A_ R—the gamma-aminobutyric acid A receptor.

Gabazine, a GABA_A_ blocker, did not affect cellular viability when administered during OGD (48.1% ± 21.6%, *n* = 34, eight cultures, Gabazine T1). However, gabazine treatment post-exposure decreased cellular viability from 49.6% ± 20.7% (*n* = 37, eight cultures, OGD with no treatment) to 35.5% ± 12.1% (*n* = 24, four cultures, Gabazine T2) (Figure 4). Viability of sham wells, which were kept for 2 h in normoxia and treated with Gabazine at T1 and T2, was 102.6% ± 5.9% (*n* = 21, seven cultures) and 100.1% ± 1.8% (*n* = 9, two cultures), respectively (Figure 4). After two-way analysis of variance, we report a significant main effect of OGD (F (1,151) = 427.0, *p* < 0.0001), but no significant effect of gabazine (F (2,151) = 2.423, *p* = 0.092) and no significant OGD–gabazine interaction (F (2,151) = 2.016, *p* = 0.136). However, Tukey’s multiple comparison test reports a significant difference between both OGD and OGD + Gabazine T2 (mean difference = 14.02, 95% CI = (2.467; 25.57), *p* = 0.0078) and OGD + Gabazine T1 and OGD + Gabazine T2 (mean difference = 12.59, 95% CI = (0.8345; 24.34), *p* = 0.0281). There was no significant difference between OGD and OGD + Gabazine T1 or between any of the normoxic treatment conditions.

### 3.4. Enhancing GABA_A_ Activation Using Isoguvacine Does Not Influence Cellular Viability Either during Oxygen-Glucose Deprivation or Post-Exposure

**Figure 5:**
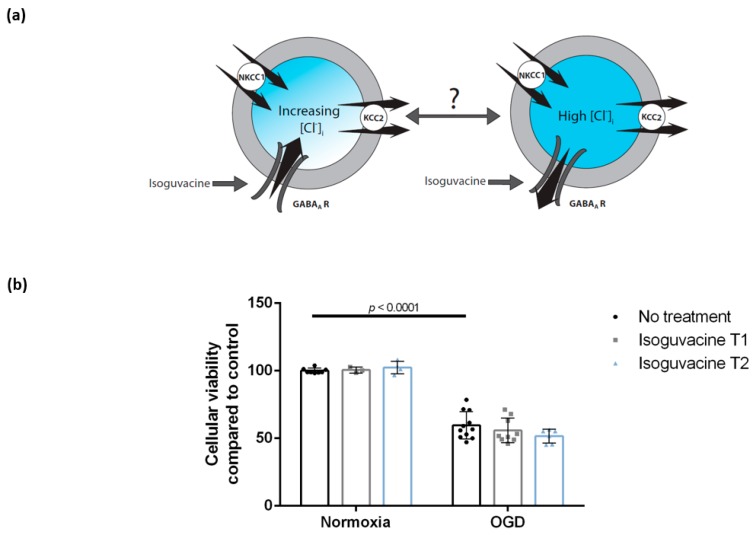
Isoguvacine treatment does not influence the viability of DIV 7 hippocampal neurons either during OGD or post-exposure. (**a**) Hypothesis: The direction of Cl^−^ transmembrane flow following GABA_A_ receptor activation depends on [Cl^−^]_i_ and its reversal potential. Enhancing GABA_A_ receptor activation with isoguvacine during or following OGD can therefore either increase the inflow or the outflow of chloride ions. (**b**) Cellular viability of DIV 7 hippocampal neurons decreased after 2 h of OGD. Treatment with the GABA_A_ agonist isoguvacine did not further influence neuronal viability. Bars represent mean ± SD. OGD—oxygen-glucose deprivation, NKCC1—the Na^+^/K^+^/Cl^−^ co-transporter, KCC2—the K^+^/Cl^−^ co-transporter, GABA_A_ R—the gamma-aminobutyric acid A receptor.

Cellular viability after OGD exposure with no treatment was 59.6% (± 10.1, *n* = 11, two cultures) and slightly lower at T1 and T2 after isoguvacine treatment (55.9% ± 9.1, *n* = 9, two cultures and 51.6% ± 5.1, *n* = 6, two cultures, respectively). Viability of sham wells, which were kept for 2 h in normoxia and treated with isoguvacine at T1 and T2, was 100.6% ± 2.2 (*n* = 3, 1 culture) and 102.4% ± 4.7, (*n* = 4, two cultures), respectively (Figure 5). There was a significant effect of OGD (F (1,35) = 312.3, *p* < 0.0001), but no significant effect of isoguvacine (F (2,35) = 0.4831, *p* = 0.6209) and no significant OGD–isoguvacine interaction (F (2,35) = 1.579, *p* = 0.2204). After Tukey’s multiple comparisons, we report no significant difference between OGD and OGD with administration of isoguvacine treatment at T1 or T2 or between any of the normoxic treatment conditions.

## 4. Discussion

In this study we explored the effect of pharmacologically blocking chloride membrane transport via the NKCC1 and KCC2 co-transporters and the effect of GABA_A_ receptor inhibition or activation on post-ischemic neuronal viability, when applied either during OGD or post-exposure. 

[Cl^−^]_i_ has been shown to increase during and after excitotoxic insult (i.e., OGD, glutamate) in several *in vitro* models: cultured cortical neurons [35], hippocampal slices [19,22], mouse organotypic hippocampal slice cultures [36]. This rise in [Cl^−^]_i_ has been directly linked to neuronal death pertaining to ischemic episodes [37] due to either cell swelling [35,38,39] or inhibitory to excitatory GABA_A_ switch [22,38]. However, the pathophysiology of chloride’s homeostatic disturbance is yet elusive.

### 4.1. Treatment with Bumetanide Is Associated with Increased Cellular Viability of DIV 7 Hippocampal Cells after Oxygen-Glucose Deprivation

Our results show that bumetanide treatment exhibits a neuroprotective effect when added during the OGD insult, but has no benefit on neuronal viability when added post-exposure. These findings extend previous studies on cultured cortical neurons suggesting that NKCC1 contributes to glutamate mediated excitotoxicity, its blocking being ineffective in preventing cell death when added after OGD exposure [35]. The underlying mechanism is thought to be the prevention of cell swelling secondary to Na^+^ and Cl^−^ entry via NKCC1, which is prevalent during the rapidly triggered glutamate-mediated excitotoxicity in early OGD, thus rendering NKCC1 a valuable therapeutic target during, but not after, OGD episodes [35,38]. Moreover, N-methyl-D-aspartate receptor activation and high extra-cellular K^+^, factors that are known to participate in ischemic damage and excitotoxicity, have been reported to stimulate NKCC1 activity in neurons [40,41,42]. There is also compelling evidence suggesting that the activity of NKCC1 increases through phosphorylation of threonine residues of its structure during ischemia, its expression remaining unchanged [19,22,39,43]. This increased NKCC1 activity creates a functional phenotype resembling the immature state where bumetanide has been initially reported to have a neuroprotective effect during OGD, in both neonatal rat hippocampal slices [44] and immature primary hippocampal cultures [24].

### 4.2. Treatment with DIOA Is Associated with Decreased Cellular Viability of DIV 7 Hippocampal Cell Cultures after Oxygen-Glucose Deprivation

DIOA treatment was detrimental when added during OGD. This effect could be explained by the supplementary increase in intracellular Cl^−^ concentration following the blockage of its outward transport, since KCC2 is one of the primary Cl^−^ extruders in mature neurons [45,46]. However, no viability changes were noted when DIOA was added during reoxygenation. These results are supported by literature data showing KCC2 protein levels significantly dropping by 30% at 1 h post OGD and further dropping by 70% at 2 h post OGD, in hippocampal slices [19]. Thus, KCC2 availability during reoxygenation might be too low for DIOA treatment to give rise to any significant changes in neuronal viability. All this data point to a narrow window of opportunity for DIOA action at the beginning of OGD, in accordance with our results. Furthermore, KCC2 has been shown to serve two main functions in mature neurons: maintaining a low [Cl^−^]_i_ to allow Cl^−^ influx via ligand-gated Cl^−^ channels, and buffering of external K^+^ concentration [47,48]. Concerning the latter function, it has been shown that, under conditions that mimic ischemia/OGD, KCC2-mediated transport will reverse in response to small increases in extracellular K^+^, generating a Cl^−^ influx [39,47,49].

### 4.3. Blocking of the GABA_A_ Receptor Using Gabazine during Reoxygenation, but Not during Oxygen-Glucose Deprivation, Is Associated with Decreased Cellular Viability of DIV 7 Hippocampal Cells

GABA release during OGD has been hypothesized to be either neuroprotective, because of its hyperpolarizing properties counteracting the glutamate-mediated excitotoxicity, or neurotoxic, due to GABA_A_ receptors’ activation facilitating Cl^−^ entry into neurons and consequent cell swelling [19,50]. We found that blocking GABA_A_ with gabazine during OGD in primary hippocampal cultures elicits no changes in cellular viability. In hippocampal slices, OGD triggers an increased glutamate and GABA release through two sequential mechanisms: exocytosis followed by a brisk anoxic depolarization and reversal of the glutamate/GABA transporters [38,51]. The anoxic depolarization is accompanied by a significant disruption of ionic homeostasis. There is a sharp increase in [Ca^2+^]_i_, which causes the inactivation of GABA_A_ receptors and subsequently prevents Cl^−^ influx during OGD [38,52,53]. This is a long-lasting inactivation, reported to persist over 1 h for high-calcium loads [53]. Other ischemia-related mechanisms involved in GABA_A_ receptor activity reduction and desensitization, such as generation of eicosanoids and reactive oxygen species or ATP level reduction, have also been described [54].

Our data is in accordance with these previous findings, as it shows that application of GABA_A_ antagonist gabazine during OGD does not significantly affect cellular viability, most likely due to the already diminished GABA_A_ activity which naturally occurs during ischemic episodes [38].

Additionally, our findings indicate that blocking GABA_A_ during reoxygenation is detrimental. Beyond the 3 h reoxygenation period, GABA_A_ inactivation gradually diminishes as ionic homeostasis is presumably restored in surviving neurons, allowing for GABA_A_ chemical manipulation. The high [Cl^−^]_i_ following OGD [19,22,35,36] is expected to cause Cl^−^ outflow, as the Cl^−^ equilibrium potential (E_Cl_) is markedly shifted towards positive values [55,56,57,58]. However, literature data suggests that the inhibitory to excitatory shift would require that E_Cl_ exceeds the membrane potential for spiking, which only occurs in severe ischemic insults [54]. Thus, most studies in animal and *in vitro* models of ischemia report neuroprotective effects of GABAergic drugs [59,60,61]. Our results support these effects, by showing that the blocking of these neuroprotective mechanisms has detrimental effects. 

### 4.4. Enhancing GABA_A_ Activation Using Isoguvacine Does Not Influence Cellular Viability Either during Oxygen-Glucose Deprivation or Post-Exposure

Inducing sedation by enhancing the activity of the GABA_A_ receptor is quite common in stroke patients and it is only sensible to ask whether this pharmacological manipulation could potentially have any detrimental effects on neuronal viability in this setting, especially since literature data are controversial regarding this matter [62,63,64]. It has also been suggested that benzodiazepines may be neuroprotective when given soon after stroke, but harmful when administered during late recovery [63]. We did not find a significant influence of GABA_A_ receptor activation with isoguvacine on cellular viability of neurons during either OGD or post-exposure.

## 5. Conclusions

In conclusion, we showed that the timing of treatment application in relation to the moment of the ischemia-reoxygenation is of paramount importance: the function of chloride co-transporters NKCC1 and KCC2 has a decisive impact on neuronal viability during the acute ischemic episode, while the GABA_A_ receptor plays a key role in the recovery period that follows. Our findings highlight a temporal widow for pharmacological modulation of chloride membrane transport as a promising neuroprotective strategy.
